# Twelve-week treadmill endurance training in mice is associated with upregulation of interleukin-15 and natural killer cell activation and increases apoptosis rate in Hepa1-6 cell-derived mouse hepatomas

**DOI:** 10.1590/1414-431X2023e12296

**Published:** 2023-08-14

**Authors:** Zhe Wang, Yunlong Cui, Yong Zhang, Xinghao Wang, Jing Li, Jialin Li, Ning Jiang

**Affiliations:** 1 Tianjin Key Laboratory of Exercise Physiology and Sports Medicine, Institute of Sport, Exercise & Health, Tianjin University of Sport, Tianjin, China; 2 Department of Hepatobiliary Surgery, Tianjin Medical University Cancer Hospital, Tianjin, China; 3 Department of Common Subject, College of Basic Sciences, Logistics College of Chinese People’s Armed Police Force, Tianjin, China

**Keywords:** Liver cancer, Natural killer cell, Apoptosis, Treadmill exercise, IL-15

## Abstract

Regular exercise reduces the risk of malignancy and decreases the recurrence of cancer. However, the mechanisms behind this protection remain to be elucidated. Natural killer (NK) cells are lymphocytes of the innate immune system, which play essential roles in immune defense and effectively prevent cancer metastasis. Physical exercise can increase the activity of NK cells. Interleukin-15 (IL-15) is the best-studied cytokine activator of NK cells, and it was shown to have many positive functional effects on NK cells to improve antitumor responses. The aim of this study was to clarify the possible important mechanisms behind endurance exercise-induced changes in NK cell function, which may be highly correlated with IL-15. An animal model was used to study IL-15 expression level, tumor volume, cancer cell apoptosis, and NK cell infiltration after treadmill exercise. Although IL-15 was highly expressed in skeletal muscle, treadmill exercise further elevated IL-15 levels in plasma and muscle (P<0.05). In addition, tumor weight and volume of tumor-bearing mice were decreased (P<0.05), and liver tumor cell apoptosis was increased after 12 weeks of treadmill exercise (P<0.05). NK cell infiltration was upregulated in tumors from treadmill exercise mice, and the level of interferon-gamma (IFN-γ) and IL-15 were higher than in sedentary mice (P<0.05). The study indicated that regular endurance training can reduce cancer risk, which was related to increased IL-15 expression, activation of the immune killing effect of NK cells, and promotion of tumor cell apoptosis, which can ultimately control tumor growth.

## Introduction

Exercise has a profound effect on the normal functioning of the immune system, and benefits of regular moderate intensity exercise for cancer patients are becoming increasingly more evident (1). Epidemiological studies have demonstrated that regular exercise reduces the risk of certain tumors and lowers the rate of tumor recurrence, which might be related to the activation of the tumor immune response (2). However, the specific mechanism of action remains to be further investigated.

Exercise has been shown to modulate the cellular immune system. During exercise, cytotoxic immune cells are mobilized into the circulation by mechanisms involving blood flow-induced shear stress as well as adrenergic signaling (3). Immune recognition and elimination of tumor cells are strong intrinsic arms in the fight against cancer, and the immune status of a tumor is accordingly closely linked to the prognosis of cancer, in which high levels of infiltrating natural killer (NK) cells and cytotoxic T cells in tumors are linked to a better prognosis of cancer patients (4). Several studies have indicated that NK cells are involved in the immuno-surveillance of cancers (5), which lyse cells through innate immune-mediated modulation of intracytoplasmic azurophilic granules. Proinflammatory cytokines modulate other immune cells directly, such as interferon (IFN)-γ and tumor necrosis factor (TNF)-α (6). It is documented that physical exercise, such as voluntary wheel running in mice, can decrease tumor growth through an exercise-dependent mobilization and redistribution of cytotoxic immune cells, in which increased levels of immune-attractant chemokines, NK cell-activating receptor ligands, and immune check-point blockade ligand were observed after exercise (7). Although exercise-mediated mobilization of immune cells was only investigated in one study with cancer patients, it was highly convincing in proving that breast cancer survivors were able to mobilize NK cells into the circulation to the same degree as age-matched healthy controls (8).

The immunological competence of NK cells is regulated by the balance between receptor signal activation and inhibition (9). Typically, the stimulatory lectin-like Nkg2d receptor is expressed on most types of NK cells. There are many ligands to Nkg2d, including MIC in most mammals and RAE-1 protein family in mice, and their integration can activate the anti-tumor effects of NK cells. Although many factors that promote NK cell maturation have been tested, interleukin-15 (IL-15) seems to be the most reasonable choice for promoting NK cell development and maturation (10-12). Moreover, several clinical trials are currently using continuous infusion of intravenous monomer IL-15 or IL-15 complex to enhance NK cell amplification (13), but its application was limited due to the difficulty of controlling the therapeutic dose and side effects (14).

Previous studies indicated that IL-15 is expressed in multiple organ tissues and is highly expressed in skeletal muscle. Therefore, IL-15 was considered a myokine and a potential effector molecule for treadmill exercise-induced benefits in immune activation. For example, Yang et al. (15) evaluated the effects of physical exercise on IL-15 expressions in skeletal muscle and adipose tissue of obese rats after a high-fat diet. Rinnov et al. (16) investigated the expression of IL-15 in skeletal muscle of humans after endurance exercise on a cycle ergometer. Hence, it is speculated that the release of IL-15 after long-term regular exercise is involved in the targeted recognition of NK cell maturation and activation of tumor cell apoptosis signals. In the present study, we aimed to further investigate the potential of IL-15 release on tumor inhibition.

## Material and Methods

### Animal care

All animal care and experiment protocols followed the regulations on animal management of the Ministry of Health of the People's Republic of China and the guidelines for the care and use of experimental animals in critical sports physiology and sports medicine experiments of Tianjin (approval No. PIFM-2019071). Beijing Fukang Biotechnology Co., Ltd. (China) provided the 7-month-old male mice. Major components in the animal food were 10% moisture, 18% crude protein, 4% crude fat, 5% crude fiber, 8% crude ash, 1.0-1.8% calcium, and 0.6-1.2% phosphorus. All the experimental mice had free access to food and water and were maintained in a specific pathogen-free environment. The animals were housed in cages separately, in a room at 22±2°C on a 12-h light/dark cycle. Forty 7-month-old male C57BL/6 mice (27±5 g) were randomly assigned to four groups (n=10 for each group) for intervention: Control (C), Treadmill exercise (T), Control Hepa1-6 (CH), and Treadmill exercise Hepa1-6 (TH).

### Animal training program

Exercise training was performed in T and TH groups before establishing tumor models. At the beginning of the experiment, mice in the T and TH groups were running on a small animal treadmill for adaption, gradually increasing acceleration using the treadmill's movement time. As the animals were housed in a cyclical environment with 12 h of light (6:00-18:00) and 12 h of darkness (18:00-6:00), the animals were uniformly trained starting at 16:00 under bright light conditions, thus avoiding disturbances to the circadian rhythm of mice. The width of each runway was 9.5 cm, and 3 mice were placed in each runway for each exercise at the same time (Beijing Zhishuduobao Biological Technology Co., Ltd., model number DB030, China). A 12-week formal exercise training was conducted after one week of adaptation training. The mice were subjected to a 60-min/day, 5-day/week exercise at a speed of 12 m/min during the light phase (17). Tired mice were encouraged to continue their training by tapping their tails and pushing them back gently. In this study, each two mice were housed in a 26 (L) × 17 (W) × 16 cm (H) cage with bedding, cage lid, food, and water bottle. The actual space for the mice to move was about 15 (L) × 14 (W) × 10 (H), and the active bottom area was 210 cm^2^. The purpose was to limit the physical activity of mice by reducing the space for mice to move.

### Liver tumor in mice

Mice were anesthetized by intraperitoneal injection of sodium pentobarbital (0.3%, 0.2 mL/10 g). Tumor models were established by injecting 100 μL Hepa1-6 (ATCC^®^ CRL-1830™) suspension (2×10^6^ cells) into the liver of CH and TH mice (18). The Hepa1-6 tumor model in C57L/J mice was chosen because it shows reliable syngeneic host growth. Hepa1-6 cells are a derivative of the BW7756 mouse hepatoma that arose in a C57L mouse. The expression of MHC class I and II is identical in C57L and C57L/J mice. The same amount of saline (0.9% NaCl) was injected into mice of the C and T groups. Pre-experimental results demonstrated that the expected experimental requirements were met on day 13 after Hepa1-6 injection. The mice in each group were sacrificed by cervical dislocation after anesthesia. This model was based on the experimental methods reported by Kröger et al. (19).

In addition, we confirmed that tumor burden did not exceed recommended levels and that animals were anesthetized and sacrificed using acceptable methods/techniques. All animal experiments were approved and supervised by the Ethics committee of Tianjin University of Sport.

### Hepatocyte isolation and flow cytometry

Isolation of primary hepatocytes was done according to published methods (20). Hepatocytes were preserved in a DMEM medium containing 10% bovine serum. NK cell surface antigen marker NK1.1 (PE Mouse Anti-Mouse NK-1.1, BD557391) was detected by flow cytometry (BD FACSCalibur, USA).

### Enzyme-linked immunosorbent assay (ELISA)

The tissue sample was ground and centrifuged at 12,000 *g* for 15 min at 4^o^C to collect the supernatant, and the protein concentration was determined by the Bradford method. The concentration of IL-15 and IFN-γ in tissue was determined by a highly sensitive enzyme-linked immunosorbent assay.

### Real-time PCR

Total RNA was extracted from liver tissue by the Trizol method. The purity and concentration of RNA were identified by an ultraviolet spectrophotometer (NanoDrop 2000, Thermo Fisher Scientific, USA). The expression of Nkg2d and Rae-1 (Applied Biosystems 7500, USA) was detected using an RT-PCR kit (One Step Sybr^®^ Primescript™ Rt-PCR kit, Takara, No. RR086A, USA). The relative expression of *Nkg2d* and *Rae-1* was calculated by the 2-ΔΔCt method. The following primers were used: *Nkg2d*: Forward: 5′-GCACTAACTACCAGTCAACCTG-3′, Reverse: 5′-GCACTAACTACCAGTCAACCTG-3′; *Rae-1*: Forward: 5′-TTTGGGAGCACAACCACAGAT-3′, Reverse: 5′-TAAAGTTGGCGGGCTGAAAGA-3′. *β-Tubulin*: Forward: 5′-AGGTCGGTGTGAACGGATTTG-3′, Reverse: 5′-GGGGTCGTTGATGGCAACA-3′.

### Western blotting

The liver tissue was homogenized in a lysis buffer containing a protease inhibitor. Protein samples were separated by sodium dodecyl sulfate-polyacrylamide gel electrophoresis (SDS-PAGE) and transferred to PVDF membranes. The membrane was exposed to the primary antibody solution (caspase-3, 1:1000, Abcam (UK); cleaved caspase-3, 1:100, Abcam; β-TUBULIN, 1:2000, Abcam) for incubation at 4°C overnight, and then exposed to secondary antibody working solution (goat anti-rabbit IgG, 1:2000, Nakasugi Jinqiao (China); goat anti-mouse IgG, 1:2000, Nakasugi Jinqiao) for 1 h at room temperature. The bands were visualized with Immobilon Western Chemilum-HRP Substrate (Millipore, USA), and the band intensity was quantified using the Image Lab (Bio-Rad, USA) software. β-tubulin was used as an internal control.

### Statistics

The data were analyzed by SPSS software (version 22.0, IBM, USA). The data are reported as means±SD of three independent experiments. Unpaired Student's *t*-test was used to compare 2 groups. Multi-group comparisons of the means were carried out by one-way analysis of variance (ANOVA) with *post hoc* comparisons by Student-Newman-Keuls test when comparing 3 groups or less. For more than 3 groups, Tukey's test was conducted for *post hoc* comparisons. Student's *t*-test was used for two groups comparisons. Statistical significance for all tests was set at P less than 0.05.

## Results

### Treadmill exercise decreased Hepa1-6 liver tumors *in vivo*

**Figure 1:**
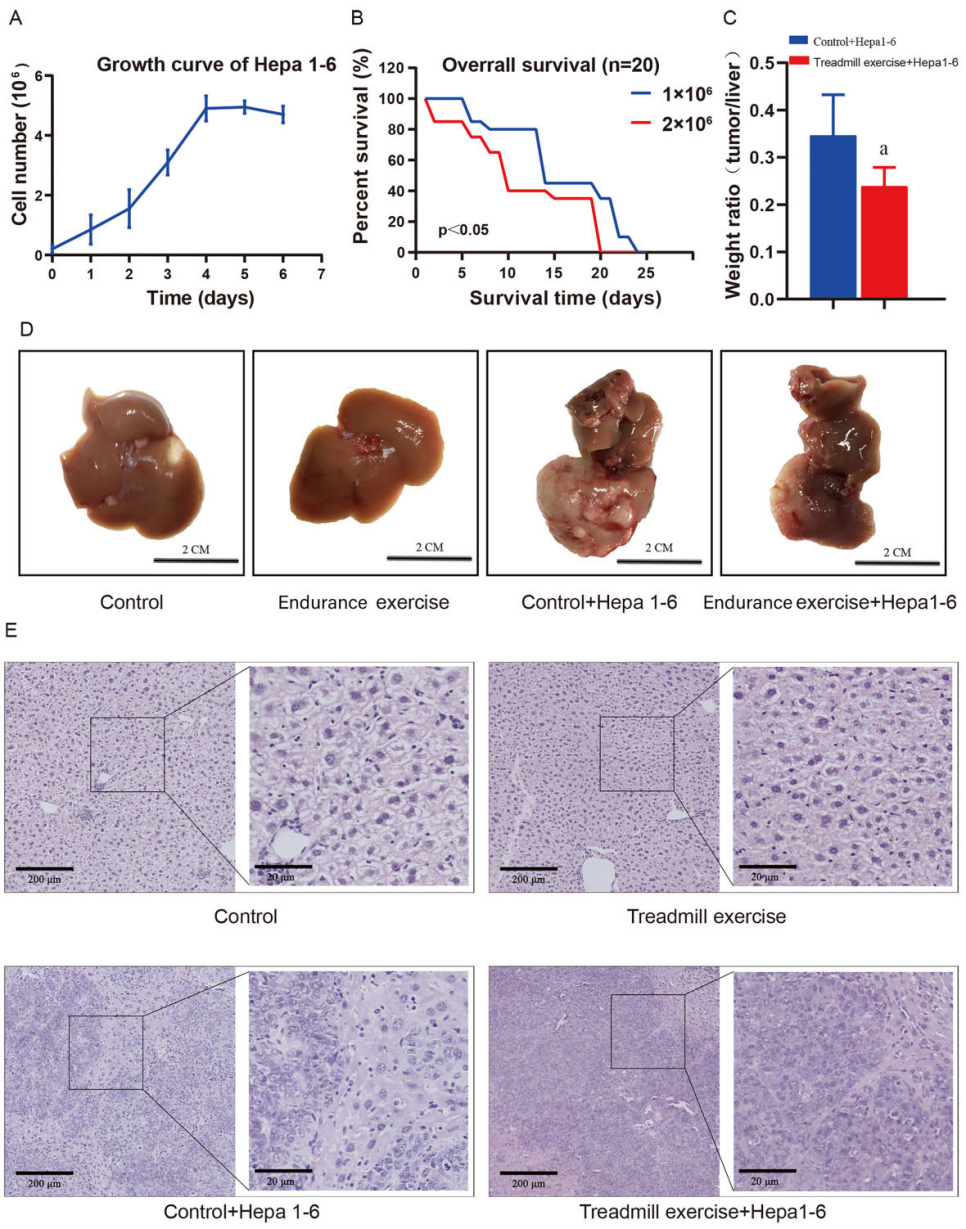
Construction of a mouse liver tumor model. **A**, Hepa1-6 cell growth curve, logarithmic growth phase was on the third day. **B**, Survival rate of mice injected with 1×10^6^ and 2×10^6^ Hepa1-6 cells. **C**, Weight ratio (tumor/liver). Data are reported as means±SD. ^a^P<0.05 *vs* control (*t*-test). **D**, Photos of the liver of mice in each group on the 13th day of inoculation of 2×10^6^ Hepa1-6 cells (scale bar 2 cm). **E**, HE stained photos of mice in each group on the 13th day of inoculation with 2×10^6^ Hepa1-6 cells (scale bars 200 and 20 μm (inset)).

The results showed that the logarithmic growth phase of Hepa1-6 was the third day after passage (Figure 1A), and the mouse liver tumor model was the most suitable on the 13th day after liver *in situ* inoculation, when 2×10^6^ Hepa1-6 cells were used for obtaining materials (Figure 1B). Treadmill exercise decreased the weight ratio of liver tumors (P=0.037) (Control: 0.341±0.02; Treadmill exercise: 0.235±0.01) (Figure 1C). The liver tissue was intact, firm, and rigid in the saline injection group without unusual findings. However, the liver tissue of the Hepa1-6 inoculation group was abnormal in morphology and rough in texture, accompanied by the appearance of white neoplastic plaques (Figure 1D). The hematoxylin-eosin (HE) staining results showed that, compared to control, the Hepa1-6 inoculation group had a greater number of tumor cells and inflammatory cells infiltrating the liver cell interstitium, the cell interstitium distribution was very uneven, the cell boundary was blurred, the nucleus had an obvious distortion, and the cell arrangement was abnormal (Figure 1E). Overall, the results showed that the *in situ* vaccination of the Hepa1-6 model was successful, and the 12-week treadmill exercise attenuated the weight of the tumor in the liver.

### Treadmill exercise attenuated Hepa1-6 development in the liver by enhancing NK cells via enhancing IL-15-related pathways

**Figure 2:**
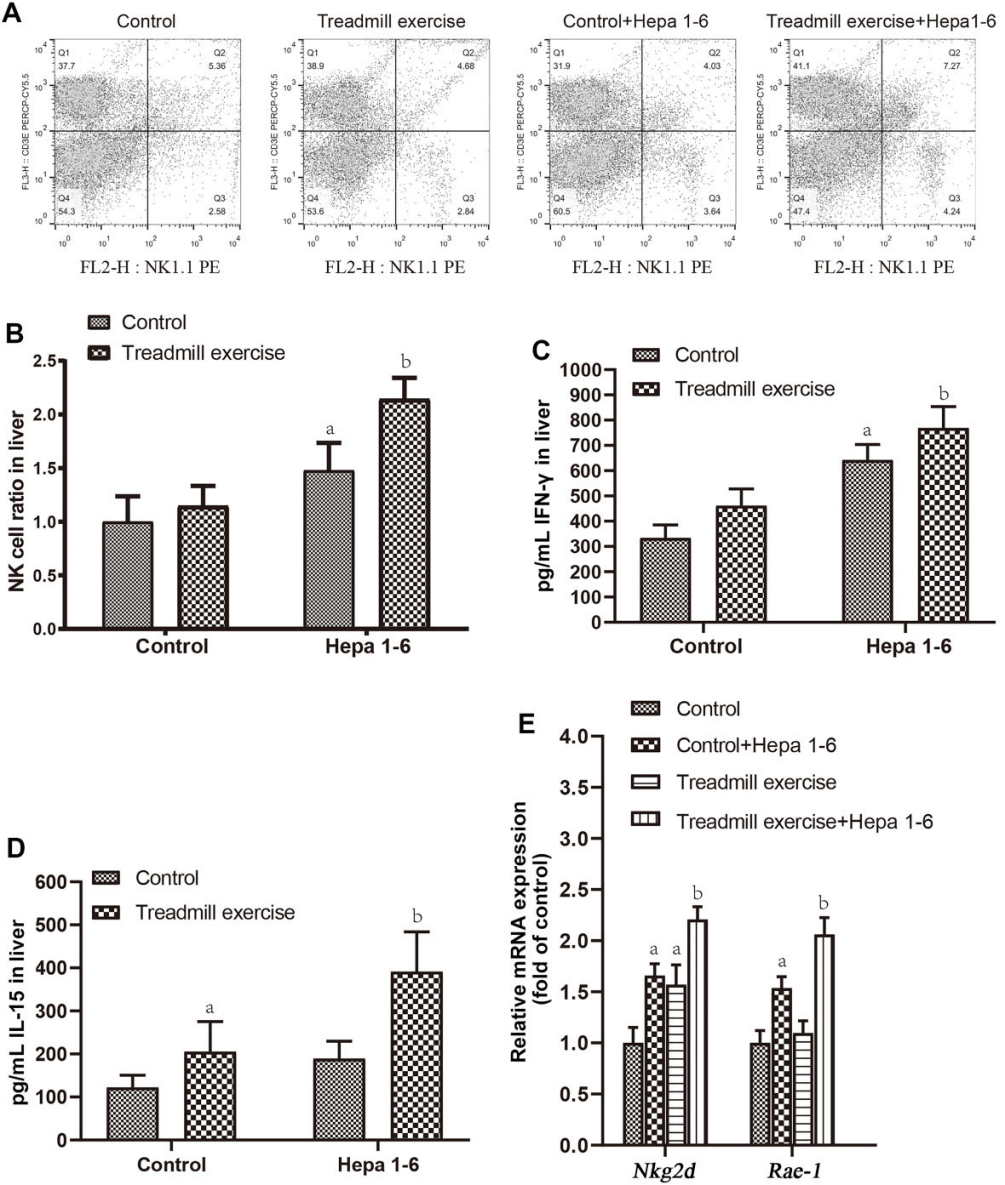
Interleukin (IL)-15 enhanced the killing effect of natural killer (NK) cells. **A** and **B**, Flow cytometry results of NK cells in liver tissue of mice in each group. **C** and **D**, ELISA results of the expression of interferon (IFN)-γ and IL-15 in the liver of each group of mice. **E**, Real-time PCR results of mRNA levels of *Nkg2d* and *Rae-1*. Data are reported as means±SD. ^a^P<0.05 *vs* control; ^b^P<0.05 *vs* control Hepa1-6 (ANOVA).

The flow cytometry results showed that the NK cell content in the liver of mice in the treadmill exercise group had no significant change (P=0.075) compared with the control group. The NK cell content in the liver of mice in the control Hepa1-6 group was increased (P=0.0004). The NK cell content in the liver of mice in the treadmill exercise Hepa1-6 group was increased (P=0.0128), especially compared with the control Hepa1-6 group (Figure 2A and B). The ELISA results showed that treadmill exercise increased IL-15 expression (P=0.0021), but IFN-γ expression did not change (P=0.6832) compared with the control group. The levels of IFN-γ and IL-15 in the hepa1-6 group were increased (P=0.0002) (Figure 2C and D) compared with the control Hepa1-6 group. RT-PCR results showed that treadmill exercise increased the mRNA expression of *Nkg2d* in the liver (P=0.0053), but the level of *Rae-1* mRNA did not change (P=0.0569) compared with the control group. The mRNA expression of *Nkg2d* and *Rae-1* in the Hepa1-6 group was increased (P<0.00001) compared with the control Hepa1-6 group (Figure 2E).

### Treadmill exercise increased apoptosis in Hepa1-6 mice liver by caspase-3

**Figure 3:**
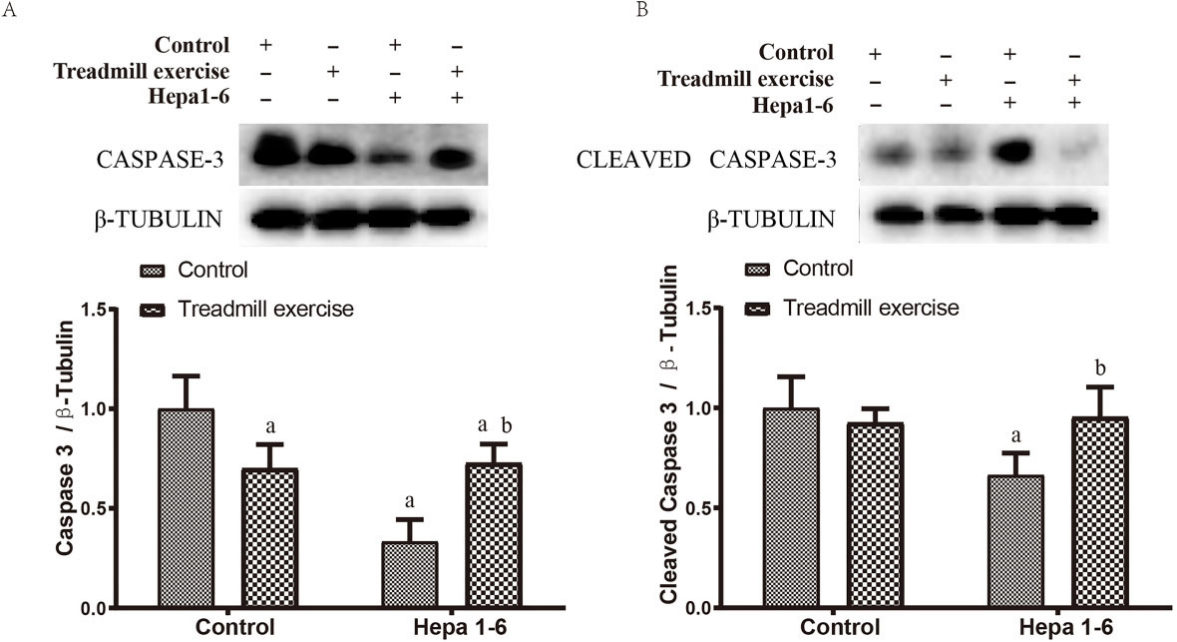
Effects of Hepa1-6 inoculation on apoptosis after treadmill exercise. Protein expression levels of caspase-3 (**A**) and cleaved caspase-3 (**B**) analyzed by Western blot. Data are reported as means±SD. ^a^P*<*0.05 *vs* control; ^b^P*<*0.05 *vs* control Hepa1-6 (ANOVA).

Western blot results showed that treadmill exercise significantly reduced the expression of caspase-3 (P<0.05), and the expression of caspase-3 and cleaved caspase-3 in the liver of Hepa1-6 mice was decreased (P<0.05) compared with the control group. However, the expression of caspase-3 and cleaved caspase-3 was increased in the treadmill exercise Hepa1-6 group (P<0.05) compared with the control Hepa1-6 group (Figure 3).

## Discussion

**Figure 4:**
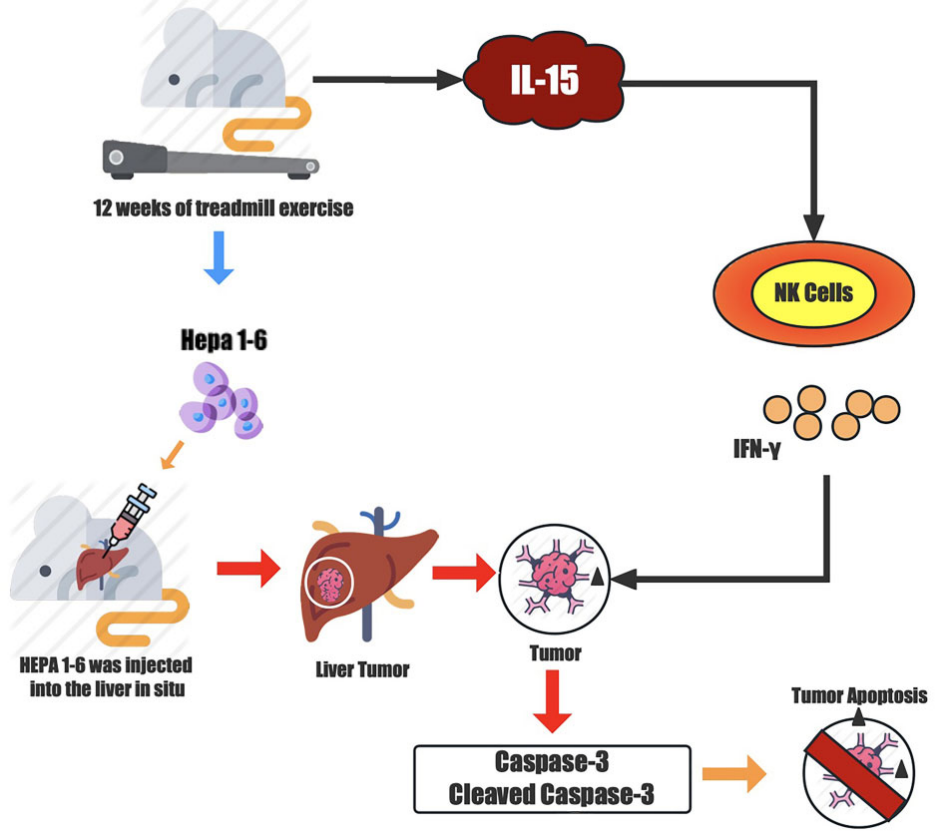
Study hypothesis. Twelve weeks of treadmill exercise activated the caspase-3 apoptosis signal of liver cells in the tumor group and delayed infection of liver tumor cells. The activation of apoptotic signals in liver tumors might be closely related to the enhancement of the killing activity of NK cells mediated by IL-15.

The present study assessed if 12 weeks of endurance exercise could delay the invasion of tumor cells and increase the expression of NK cells in mice. Yang et al. (21) proved that IL-15 is key to NK cell maturation. NK cells are essential for tumor immunity as they have the unique ability to distinguish tumor cells from normal cells (22). Based on our findings, we propose that the increase of IL-15 in plasma caused by treadmill exercise plays a vital role in mediating NK cell recognition and killing the tumor (Figure 4). The results showed that in the tumor model, the level of IL-15 in plasma in pre-trained mice was significantly increased, which enhanced the identification and killing ability of NK cells. Moreover, the caspase-3 apoptosis pathway in the liver tumor tissue of mice was also activated. However, whether a causal relationship between the three exists still needs further study.

As founding members of the innate lymphocyte family, NK cells have recently been recognized as effective in controlling microbial infection and tumor progression. In patients and animal models, impaired NK cells or NK cell deficiency could increase the incidence of various types of cancer (23). NK cells were shown to play a role by not being separated from the surface and inhibiting receptor activation by activation of the receptor-targeted killing of abnormal cells (24). Nkg2d, also known as CD314 and KLRK1, was the most important immune monitoring receptor on the surface of NK cells (25), which was expressed by all NK cells, CD8+ T cells, and γδT cells. Certain viruses and tumor cells develop immune escape mechanisms by bypassing Nkg2d to mediate immune monitoring. NK cells cannot be recognized due to the loss of ligands on the tumor cell's surface, demonstrating the importance of NK by ligand-mediated immune response (25). Nkg2d ligands of humans are mainly MHC class I polypeptide-related sequence A (MICA), MICB, retinoic acid early transcript 1E protein (Raet1E), Raet1G, Raet1H, Raet1I, Raet1L, and Raet1N, whereas, in mice, its ligands are the RAE1 family (22). These ligands were not present in most healthy tissue cell surfaces, but they affect infected and tumor cells (25). Mature NK cells can release perforin and granzyme (e.g., IFN-γ, TNF-α, GCSF, etc.) through the combination of receptor and ligand and death receptor (such as FAS) acting on target cells, which activates the caspase enzymatic cascade reaction (26). Wang and Weng (27) showed that NK cells and Nkg2d were significantly increased after exercise compared with the control group in young men. Remarkably, the number of NK cells gradually decreased in 24 h. However, the Nkg2d remained at a high level (27). In our results, exercise training increased the expression of NK cell ligands *Nkg2d* and *Rae-1* in liver tissue of tumor-bearing mice, and the content of IFN-γ was increased. Thus, exercise training can enhance the killing ability of NK cells.

IL-15 is an essential factor for NK cell maturation. Clinical trials and animal models have achieved positive results by injecting IL-15 to treat cancer. Yang et al. (21) suggest that IL-15 act by activating the *e4bp4* gene, thereby promoting differentiation and maturation of NK cells, confirming the importance of IL-15 for mature NK cells. IL-15 was confirmed clinically to enhance immunotherapy in several conditions (28). Conlon et al. (13) showed that when recombinant human IL-15 was given to patients with metastatic malignant tumors, IL-15 could alter the homeostasis of lymphocyte subsets in blood and increase the content of NK cells significantly. In another study, a transgenic mouse that overexpresses IL-15 by eliminating posttranscriptional checkpoints was used; NK cells and T cells increased robustly, causing fatal lymphocytic leukemia (29). Despite the advantage of IL-15 to promote NK cell maturation, there are many uncertain negative factors for IL-15. Therefore, the application perspective of IL-15 is still unclear.

Skeletal muscle, as an endocrine organ, has paracrine or endocrine functions. Skeletal muscle expresses several myokines to participate in metabolic regulation. Nieman et al. (30) showed that IL-15 was the most highly expressed cytokine measured at the mRNA level in human skeletal muscle. Molanouri Shamsi et al. (31) found a significant increase in serum IL-15 concentrations due to training in healthy and diabetic rats. Immediately after exercise training, the level of IL-15 in plasma increased due to muscle contraction-induced secretion (32). Christiansen et al. (33) pointed out that exercise alone did not affect the expression of muscle IL-15 levels in an obesity model. Previous studies on the effects of exercise on IL-15 in skeletal muscle and content of IL-15 in plasma were inconsistent (16). In the present study, it was found that although exercise could not improve the expression of IL-15 in the plasma of normal mice, the content of IL-15 in the plasma of tumor-bearing mice after 12 weeks of treadmill exercise was significantly increased.

Apoptosis is considered a typical anti-oncogenic process. Apoptosis is a genetically programmed cell suicide regulated by activating a family of cysteine proteases (caspase) that mediates caspase-3, -6, and -7. Caspase-3 is a key mediator of mammalian cell apoptosis, cleaving many essential proteins. Apoptosis is transduced through the death receptor pathway. In FASL (FAS ligand) binding, the cytoplasmic tails of FAS recruit the adaptor protein FADD (Fas-associated protein with death domain), which activates caspase-8 and caspase-3 to degrade cellular components (34). The activation of the caspase family is the best recognized biochemical hallmark of early and late apoptotic stages. The detection of active caspase-3 in cells and tissues is an essential method to detect apoptosis induced by many apoptotic signals. Many anticancer therapies cause tumor cell death by activating caspase-3. Therefore, caspase-3 activation has been used by many researchers as an alternative marker for the effectiveness of cancer treatment (35). Contrarily, caspase-3 promoted the proliferation of tumors after radiotherapy through paracrine signaling pathways (36). Recent studies have shown that caspase-3 is involved in drug resistance and radiation resistance of colon cancer cells and migration, invasion, and metastasis of colon cancer cells (37). In the present study, 12-week treadmill exercise could increase the apoptosis signal of caspase-3 in liver tumors.

This article explored whether there is a correlation between IL-15, NK cells, and tumor apoptosis. To better verify this correlation, the IL-15 KO mice were established to further explain the underlying mechanisms. In the present study, compared with group C, the levels of IL-15 and *Nkg2d* in group T increased. It should be noted that we did not observe any difference in NK cells between the two groups. However, when it comes to the tumor groups, not only IL-15 and *Nkg2d* but also NK cells were significantly higher in the TH group than in the CH group. We speculated that this may be because the efficiency of NK cells in identifying and killing tumor cells was dramatically increased. In addition, the combination of *Nkg2d* and *Rae-1* mediated the activation of NK cells in the tumor model. *Nkg2d* may be the critical factor in the early warning mechanism of NK cell recognition efficiency, and we will further explore *Nkg2d* in future experiments.

In this study, we used pre-exercise training as the primary variable. We kept the tumor modeling time and dosage as consistent as possible in different groups. The relationship between pre-exercise training and survival rate of mice will be evaluated in future experiments.

### Conclusions

In conclusion, it was proven that 12 weeks of treadmill exercise activated the caspase-3 apoptosis signal of liver cells in the tumor group and delayed infection by liver tumor cells. The activation of apoptotic signals in liver tumors might be closely related to the enhancement of the killing activity of NK cells mediated by IL-15.
